# RNA marker modifications reveal the necessity for rigorous preparation protocols to avoid artifacts in epitranscriptomic analysis

**DOI:** 10.1093/nar/gkab1150

**Published:** 2021-12-01

**Authors:** Florian Richter, Johanna E Plehn, Larissa Bessler, Jasmin Hertler, Marko Jörg, Cansu Cirzi, Francesca Tuorto, Kristina Friedland, Mark Helm

**Affiliations:** Institute of Pharmaceutical and Biomedical Sciences, Johannes Gutenberg-University Mainz, Staudingerweg 5 55128 Mainz, Germany; Institute of Pharmaceutical and Biomedical Sciences, Johannes Gutenberg-University Mainz, Staudingerweg 5 55128 Mainz, Germany; Institute of Pharmaceutical and Biomedical Sciences, Johannes Gutenberg-University Mainz, Staudingerweg 5 55128 Mainz, Germany; Institute of Pharmaceutical and Biomedical Sciences, Johannes Gutenberg-University Mainz, Staudingerweg 5 55128 Mainz, Germany; Institute of Pharmaceutical and Biomedical Sciences, Johannes Gutenberg-University Mainz, Staudingerweg 5 55128 Mainz, Germany; Division of Epigenetics, DKFZ-ZMBH Alliance, German Cancer Research Center (DKFZ) 69120 Heidelberg, Germany; Faculty of Biosciences, University of Heidelberg 69120 Heidelberg, Germany; Division of Biochemistry, Mannheim Institute for Innate Immunoscience (MI3), Medical Faculty Mannheim, Heidelberg University, Mannheim, Germany; Center for Molecular Biology of Heidelberg University (ZMBH), DKFZ-ZMBH Alliance69120 Heidelberg, Germany; Institute of Pharmaceutical and Biomedical Sciences, Johannes Gutenberg-University Mainz, Staudingerweg 5 55128 Mainz, Germany; Institute of Pharmaceutical and Biomedical Sciences, Johannes Gutenberg-University Mainz, Staudingerweg 5 55128 Mainz, Germany

## Abstract

The accurate definition of an epitranscriptome is endangered by artefacts resulting from RNA degradation after cell death, a ubiquitous yet little investigated process. By tracing RNA marker modifications through tissue preparation protocols, we identified a major blind spot from daily lab routine, that has massive impact on modification analysis in small RNAs. In particular, m^6,6^A and Am as co-varying rRNA marker modifications, appeared in small RNA fractions following rRNA degradation *in vitro* and *in cellulo*. Analysing mouse tissue at different time points *post mortem*, we tracked the progress of intracellular RNA degradation after cell death, and found it reflected in RNA modification patterns. Differences were dramatic between liver, where RNA degradation commenced immediately after death, and brain, yielding essentially undamaged RNA. RNA integrity correlated with low amounts of co-varying rRNA markers. Thus validated RNA preparations featured differentially modified tRNA populations whose information content allowed a distinction even among the related brain tissues cortex, cerebellum and hippocampus. Inversely, advanced cell death correlated with high rRNA marker content, and correspondingly little with the naïve state of living tissue. Therefore, unless RNA and tissue preparations are executed with utmost care, interpretation of modification patterns in tRNA and small RNA are prone to artefacts.

## INTRODUCTION

Current intense research efforts promote a coalescence of RNA modification data into a more complete picture of what is now known as ‘the epitranscriptome’. Our knowledge concerning chemical structure, position, stoichiometry and dynamics of eukaryotic modifications in general, and of mammalian ones in particular, have entered the system-wide stage. However, in as much as the term epitranscriptome includes all post-transcriptional events, its understanding must include the arguably most widespread chemical manipulation of RNA, that is: cleavage. Hydrolysis of RNA phosphoester bonds commences directly after transcription and shapes the (epi)trancriptome all the way to nucleoside excretion or salvage, including processing steps like splicing, trimming and RNA degradation in the exosome (1–7). Artificial concepts of anabolic processing versus catabolic degradation have undergone repeated challenges in the RNA field (8,9) This holds true especially for small RNAs below ∼40 nucleotides, which were originally thought of as ‘debris’ at the bottom of PAGE gels. This fraction was subsequently discovered to contain many small RNAs of high biological relevance in sophisticated pathways, such as miRNA, siRNA, piRNAs and others (9–12). More recently, even more alleged debris from the same size fraction were shown to exhibit specific functions in the regulation of gene expression, namely fragments of tRNAs and rRNAs (9). However, in depth characterization of the remainder, the ‘RNA junk’ is still lacking, and so we still do not know the exact composition of small RNA preparations. Given the above cited discoveries in this pool of RNA species, its composition is expected to strongly depend on the metabolic state of the cell, as influenced by numerous outside stimuli. As such, its potential in gene regulation and therefore its characterization is even more interesting, but the associated problems include in particular copy number quantification, which is typically approached by RNAseq techniques involving reverse transcription. The latter, however, are greatly hampered in their quantification accuracy by RNA modifications, as evidence by numerous recent, ever more sophisticated techniques for tRNA quantification (13–16).

The above issues have several important implications for the definition of the epitranscriptome. Given the high modification density of rRNAs, and even more of tRNAs, their degradation must necessarily cause the respective modifications to end up in the small RNA fraction (17–19). In contrast to proteins, however, the relative contributions of degradation *in cellulo* versus *in vitro*, i.e. during the isolation procedure, are entirely unclear. Moving further along these lines, small RNAs are also subject to eventual turnover, and thus vanish from the small RNA fraction along with their modifications (20,21). Similar considerations apply to RNA preparations of tRNA size.

The above sketch, plausible as it may be, has not yet been systematically supported by experimental data. It does, however, bear substantial importance, given increasing numbers of reported modifications of species from the small RNA fraction. Indeed, between modification analytics by RNAseq methods and LC–MS, an important open question is the amount of contaminating rRNA fragments (22–26), and their genesis, e.g. by processing in the living cell, intracellular degradation during cell death, or unspecific degradation during RNA isolation.

In conclusion of the above, the interplay of RNA modifications and nucleolytic events is likely to strongly influence the distribution of RNA modifications in RNAs of different sizes (27,28). A preparation of total RNA, even from fresh tissue or tissue culture cannot a priori be assumed to reflect that of a homogenous population of vital and viable cells, given the RNA contributions from subpopulations of apoptotic, necrotic, or other dead or dying cells, and additional unspecific degradation contributed by the isolation protocol itself (29–33). Interestingly, studies of post-mortem RNA decay are extremely scarce (34–36).

We therefore perceive an urgent requirement for a characterization of degradation events in tissues after organismal death, in order to determine conditions under which RNA isolates would reflect the physiological state, unbiased by artificial degradation. Because of the known problems with RNAseq (*vide supra*), we decided to trace the fate of rRNA and tRNAs during degradation *via* detection of their characteristic modifications. We found that, in line with large sections of published literature, certain modifications or combinations thereof, are unique or sufficiently characteristic to a given RNA type, and allow tracing of corresponding RNA species in the course of continuous degradation to ever shorter RNA fragments. In particular, modifications typically found in tRNAs and rRNAs emerge in the small RNA fraction as a consequence of continued degradation. Pronounced differences were found among different tissues, in particular between liver and brain. As a major finding, we define conditions under which characteristic modification patterns for the distinction among different brain tissues become apparent.

## MATERIALS AND METHODS

### Animals

C57BL/6 mice for Mainz based experiments were purchased from Janvier Labs. B6.129S mice were provided by JAX (MMRRC). After delivery, mice were kept for at least 14 days in the animal facility to reduce stress associated with transport. Animals were housed in groups in standard cages. They were provided with food and water ad libitum, with paper towels as cage enrichment, and kept on a 12:12 h light:dark cycle (lights on at 0700 hours). Animals were killed by dissection according to European Communities Council Directive (86/609/EEC) and approved by the local governmental commission for animal health. Heidelberg based experiments were conducted on C57Bl/6J mice at the German Cancer Research Centre (DKFZ) according to applicable laws and regulations. Animal experiments were conducted on adult mice at age between 5–8-month-old.

### RNA preparation

#### Isolation of total RNA from mouse tissue

Mice were sacrificed using cervical dislocation. Brain areas, whole brain (without olfactory bulb and brainstem) and liver tissue were dissected. Total RNA was prepared using 1 ml TRI Reagent^®^ (SIGMA, T9424-200ml) for 100 mg tissue, followed by addition of 200 μl chloroform (Honeywell Riedel-de Haën, 34854-2.5L). After incubation (10 min) at room temperature (RT), tissue suspension was centrifuged (16 000 × g, 4°C, 15 min). The upper aqueous phase was taken and mixed in a 1:1 ratio with isopropanol (Honeywell Riedel-de Haën, 34965-4 × 2.5 l) and 1 μl glycogen (Thermo Fisher Scientific, R0551) was added, incubated at RT (5 min) and centrifuged (16 000 × g, 4°C, 5 min). The RNA pellet was washed twice with ice-cold 75% ethanol including centrifugation (16 000 × g, 4°C, 5 min). RNA was reconstituted in 50 μl nuclease-free water (Zymo Research, W1001-10). RNA concentrations were determined using UV-VIS spectrophotometer Nanodrop 2000 (Thermo Fisher Scientific, Waltham, USA) and the integrity of the RNA was checked using the Agilent TapeStation 4200 system (Agilent, Santa Clara, USA) analysis to obtain eRIN values.

#### Purification of tRNA and RF-fractions

Total RNA was further separated for tRNA and RNA fragments (RF) by 10% urea polyacrylamide gel electrophoresis (PAGE) for at least 1 h. The gel solution was mixed using a 50:40:10 ratio of Rotiphorese^®^ Sequencing gel concentrate (Carl Roth, 3043.1), Rotiphorese^®^ Sequencing gel diluent (Carl Roth, 3047.1) and Rotiphorese® Sequencing gel buffer concentrate (Carl Roth, 3050.1). The dimensions of the gels were 35 cm × 20 cm × 0.01 cm (*H* × *B* × *T*) and seven pockets with 2 cm width. 35 μg total RNA was mixed with 6× TriTrack DNA loading dye (Thermo Fisher Scientific, R1161) in a 1:3 mixture; the loaded volume did not extent 50 μl per pocket. Gels were pre-run for 30 min limited by the current to 40 mA and the power to 300 V using the Bio-Rad PowerPac™ 300 (Bio-Rad, Feldkirchen, Germany). The gel running apparatus BRL Model SA was provided by Life Technologies Inc. (Carlsbad, USA). After harvesting the gels, they were stained in aqueous ethidium bromide solution (Roth, 2218.1) for 5 min and scanned on a Fusion Pulse TS (Vilber Lourmat, Marne-la-Vallée cedex, France) with 400 ms exposure time. In order to recieve tRNAs a band between 60 and 75 nucleotides (nt) was dissected according to the ultra-low range size ladder (Thermo Scientific, SM1213). Second, a rather broad band corresponding to ∼15–50 nt was dissected and named RNA fragment (RF) fraction. RNA was eluted from the gel pieces using the ZR small-RNA™ PAGE Recovery Kit for 20 preparations (Zymo Research, R1070, Lot No.: ZRC205441) according to the manufacturers specifications. RNA Recovery Buffer (R1070-1), RNA MAX Buffer (R1070-2), RNA Prep Buffer (R1060-2), RNA Wash Buffer (R1003-3), Zymo-Spin™ IC Columns (C1004), Zymo-Spin™ IIICG Columns (C1006), Zymo-Spin™ IV Columns (C1007), Collection Tubes (C1001), DNase/RNase-Free Water (W1001). In the last step, RNA was redissolved in 20 μl of the nuclease free water (Zymo Research, W1001-1). The RNA concentration was again determined using the UV-VIS spectrophotometer Nanodrop 2000.

#### rRNA isolation

A prestained 0.8% agarose gel (Biozym, Germany) containing 1× TBE and 1× SYBR^®^Gold (Thermo Fisher Scientific, Germany) was cast using two combs, with comb #1 conventionally placed near the cathode and comb #2 placed towards the anode end of the gel. The RNA sample in 1× loading dye containing formamide (Roth, Germany) was applied to the wells left by comb #1. Progress of electrophoresis was monitored in real-time by a Blue Light Transilluminator (Dark Reader from Clare Chemical Research, USA). The electrophoresis was performed at 180 V for about 90 min until the first fraction of interest (rRNA) reached the pockets resulting from comb #2. The respective fractions were removed during their passage through the second pocket by pipetting, and precipitated after addition of ammonium acetate/ethanol (1/10 volume of 5 M ammonium acetate (Merck-Millipore, Germany), 1 μl glycogen (5 mg/ml, Thermo Fisher Scientific, Germany) and 2 vol. 100% ethanol (Carl Roth, Germany)). The samples were incubated at −80°C for 1 h or at −20°C overnight. The RNA pellet was collected after centrifugation at 12 000 g at −4°C for 45 min, washed with 75% ethanol and again centrifuged at 12 000 g at −4°C for 15 min. The resulting RNA pellet was dissolved in ultrapure water. All samples were filtered through 0.2 μm solid phase filters (Nanosep centrifugal device, Pall, USA).

#### Purification of mRNA

Purification from total RNA was performed according to (37). In brief, ∼60 μg of total RNA were first treated with DNase I (Thermo Scientific) to avoid DNA contamination before incubation with 100 μl washed oligo d(T)25 magnetic beads (New England Biolabs), to isolate mRNA from total RNA according to a modified protocol of Dynabeads (Thermo Scientific), including a second round of purification and elution in MilliQ water.

### LC−MS/MS of modified RNA nucleosides

#### Sample digestion

Up to 300 ng of tRNA or RF-RNA per sample was digested to nucleosides using 0.6 U nuclease P1 from *P. citrinum* (Sigma-Aldrich), 0.2 U snake venom phosphodiesterase from *C. adamanteus* (Worthington), 2 U FastAP (Thermo Scientific), 10 U Benzonase (Sigma-Aldrich), 200 ng Pentostatin (Sigma-Aldrich) and 25 mM ammonium acetate (pH 7.5; Sigma-Aldrich) over night at 37°C in a total volume of 30 μl. For each technical replicate (injection to the LC−MS/MS system) 150 ng of tRNA was digested. For RF-RNA at minimum 75 ng was used per injection.

#### Data acquisition

For the sample injection a mixture of the ^13^C internal standard and the sample was prepared. The internal standard dilution (ISTD) is a long-lasting stock mix of 50 ng/μl *Escherichia coli* and 50 ng/μl *Saccharomyces cerevisiae*. 1.1 μl ISTD was added per injection to the 30 μl volume accounting for the 10% of sample, that cannot be taken by the syringe of the auto sampler. All measurements were performed on an 1260 Infinity II LC (Agilent, Santa Clara, United States) coupled to an Agilent 6460 Triple Quadrupole mass detection system (Agilent, Santa Clara, CA, USA). The separation was conducted on a Synergi™ Fusion RP C18 column (S-4 μm, 80 Å, column size: 250 × 2.0 mm I.D.) from Phenomenex (Torrance, United States). Column oven was set to 35°C. Moreover a gradient method termed NUCS4 was used, defined by the following conditions. Phase A containing acetonitrile (ACN) (Honeywell Riedel-de Haën, 34967-2.5L) starting at 0% and increased in 10 min to 8% ACN. Phase B contained NH_4_OAc buffer (pH 5.3). This was followed by a steeper gradient to 40% ACN in 10 min. Equilibration time was another 10 min. The flow rate was 0.35 ml/min. 5 mM NH_4_OAc buffer was freshly prepared. More hydrophobic modifications were not included. The UV trace was recorded in a Multiple wavelength detector (MWD) detector at 254 nm at an attenuation of 1000 mAU at a sample rate of 2.5 Hz.

The sample was injected to the Mass Spectrometer via the EJS ESI Source in the positive ion mode. The following parameters were defined for the measurement: Capillary current 5400 nA, gas temperature 350°C, sheath gas temperature 345°C, sheath gas flow 10.0 l/min, gas flow 8 l/min, Nebulizer 50.0 psi, Corona voltage 0 V. MRMs were programmed with a window of 6 min around the optimized elution time for the following modifications and their ^13^C-labeled counterparts: Am, Cm, D, Gm, I, i^6^A, m^1^A, m^1^G, m^2,2^G, m^6,6^A, m^2^A, m^2^G, m^3^C, m^3^U, m^5^C, m^5^U, m^6^A, m^7^G, mcm^5^U, ms^2^i^6^A, Psi, Q, S^2^C, S^2^U, t^6^A, Tm, Um (abbreviations according to MODOMICS). Parameters of the acquisition method are given in Table 1.

**Table 1.** Details of LC−MS/MS detection of modified nucleosides

Dynamic MRM								
Compound name	Precursor ion	Product ion	Fragmentor	Collision energy	Cell accel. voltage	Ret time (min)	ΔRet time	Polarity
Am	282	136.0	92	13	2	16.4	5	Positive
Am 13C	293	141.0	92	13	2	16.4	5	Positive
Cm	258	112.1	60	9	2	10.0	5	Positive
Cm 13C	268	116.1	60	9	2	10.0	5	Positive
D	247	115.0	80	10	2	3.9	5	Positive
D 13C	255	119.0	80	10	2	3.9	5	Positive
Gm	298	152.0	72	5	2	13.2	5	Positive
Gm 13C	309	157.0	72	5	2	13.2	5	Positive
I	269	137.0	76	5	2	10.5	5	Positive
I 13C	279	142.0	76	5	2	10.5	5	Positive
i^6^A	336	204.0	80	15	2	22.3	5	Positive
i^6^A 13C	351	214.0	80	15	2	22.3	5	Positive
m^1^A	282	150.0	92	17	2	5.6	6	Positive
m^1^A C13	293	156.0	92	17	2	5.6	6	Positive
m^1^G	298	166.0	82	9	2	13.0	5	Positive
m^1^G C13	309	172.0	82	9	2	13.0	5	Positive
m^2,2^G	312	180.0	82	9	2	15.4	5	Positive
m^2,2^G 13C	324	187.0	82	9	2	15.4	5	Positive
m^6,6^A	296	164.1	102	17	2	18.9	5	Positive
m^6,6^A 13C	308	171.0	102	17	2	18.9	5	Positive
m^2^A	282	150.1	92	17	2	16.9	5	Positive
m^2^A 13C	293	156.0	92	17	2	16.9	5	Positive
m^2^G	298	166.1	82	9	2	13.6	5	Positive
m^2^G 13C	309	172.0	82	9	2	13.6	5	Positive
m^3^C	258	126.0	40	9	2	6.8	6	Positive
m^3^C C13	268	131.0	40	9	2	6.8	6	Positive
m^3^U	259	127.0	76	5	2	12.6	6	Positive
m^3^U 13C	269	132.0	76	5	2	12.6	6	Positive
m^5^C	258	126.1	40	9	2	9.5	5	Positive
m^5^C 13C	268	131.0	40	9	2	9.5	5	Positive
m^5^U	259	127.0	76	5	2	11.2	6	Positive
m^5^U 13C	269	132.0	76	5	2	11.2	6	Positive
m^6^A	282	150.1	92	17	2	17.1	5	Positive
m^6^A 13C	293	156.0	92	17	2	17.1	5	Positive
m^7^G	298	166.1	82	9	2	9.7	6	Positive
m^7^G C13	309	172.0	82	9	2	9.7	6	Positive
mcm^5^U	317	185.0	66	5	2	13.2	5	Positive
mcm^5^U 13C	329	192.0	66	5	2	13.2	5	Positive
ms^2^i^6^A	382	250.0	80	15	2	22.4	5	Positive
ms^2^i^6^A 13C	398	261.0	80	15	2	22.4	5	Positive
Ψ	245	209.0	81	5	2	4.2	5	Positive
Ψ C13	254	218.0	81	5	2	4.2	5	Positive
Q	410	163.0	80	15	2	11.6	6	Positive
Q 13C	427	170.0	80	15	2	11.6	6	Positive
s^2^C	260	128.0	40	10	2	9.2	6	Positive
s^2^C 13C	269	132.0	40	10	2	9.2	6	Positive
s^2^U	261	129.0	66	5	2	11.1	6	Positive
s^2^U 13C	270	133.0	66	5	2	11.1	6	Positive
t^6^A	413	281.0	80	15	2	15.2	8	Positive
t^6^A 13C	428	291.0	80	15	2	15.2	8	Positive
Tm	273	127.0	66	5	2	14.9	5	Positive
Tm 13C	286	134.0	66	5	2	14.9	5	Positive
Um	259	113.0	66	5	2	12.1	5	Positive
Um 13C	269	117.0	66	5	2	12.1	5	Positive

#### Data analysis

Data was extracted with the following programs: Extraction of the UV trace area was done in Agilent Mass Hunter Qualitative Analysis B.05.00 for the canonical nucleosides C, U, G and A and the average was calculated. For relative quantification, ion chromatograms of the Mass Spectrometry dynamic Multiple Reaction Monitoring (MRM) were extracted in Skyline 20.1.0.76 (MacCoss Lab, department of Genome Sciences) and allowed integration of respective peaks, providing values for the area under the curve (AUC). Only peaks with a normal distributed peak shape and a minimum signal-to-noise ratio of 5 in at least one of several compared samples were scored in further analysis (heatmaps or PCAs), while modifications with low abundance in all compared samples were excluded (shown in grey below heatmaps). Those modifications contained in the ^13^C SILIS (Table 1) were normalized as outlined below, the remainder of modifications were not corrected for ^13^C. For each modification the ratio of ^12^C and ^13^C of AUC was calculated. This value was further normalized to the C/U/G/A UV average and considering the UV signal caused by the addition of internal standard multiplied by 50 ng. To create heatmaps of tRNA- and RF-fractions, a third normalization step was applied to make changes in different peak amplitudes comparable. Therefore, the mean value from all samples for one modification was calculated and next, each sample value was shown in a relation to this mean. For absolute quantification, internal and external calibration was combined, as described previously (38).

#### Statistical analysis

Statistical procedures were applied in Perseus 1.509 Software released (Jürgen Cox and Mathias Mann, MPI Munich). Data was normalized by dividing each row by its mean for cluster analysis driven heatmaps generation using the *k*-means value and accounting for a maximum of 300 clusters. One- or two-sided clusters were used alternatively. Principle Component Analysis was performed with five components, and a *P*-value threshold of 0.05. In cases with ≥3 replicates two-sided Student's *t*-tests were performed with a *P*-value of 0.01 to account for the low number of parameters/modifications.

### Isolation of mitochondrial RNA

One male mouse (B6.129) was sacrificed using cervical dislocation at the age of 12 weeks. Whole brain (without olfactory bulb and brainstem) and liver tissue was dissected and placed in ice cold phosphate buffered saline (PBS) to rinse away blood.

In the first purification step, tissue was cut with a scalpel and then transferred into the homogenization vessel with 6 ml of Mitochondria Isolation Buffer. Homogenization was performed in a Braun Potter S Homogenizor (B. Braun Biotech International, Berlin, Germany) on ice and five passes were executed. Homogenates were transferred into a new tube and centrifuged with a fixed angle rotor (750 × g, 4°C, 10 min). The supernatant was saved in a new tube and placed on ice, while the pellet was re-suspended in 500 μl MIB and centrifuged again (750 × g, 4°C, 5 min). Both supernatants were combined before the high-spin centrifugation step (10 000 × g, 4°C, 5 min). Supernatant was discarded and the mitochondria pellet was resuspended in 500 μl MIB.

For obtaining higher purity, mitochondria were further enriched and concentrated in a discontinuous Percoll^®^ gradient (SIGMA, P1644-500ml). For this purpose, liver and brain mitochondria obtained from the first part of the protocol were resuspended in 28 ml of a 12%-Percoll^®^ dilution in MIB. This solution was then carefully applied to a two-step gradient with 7 ml 26%-Percoll^®^ in the upper layer and 4 ml 40%-Percoll^®^ in the bottom layer. For best resolution, the 28 ml brain and the 28 mL liver mitochondria were divided onto four gradient tubes each. A first ultracentrifugation step was performed in a fixed angle rotor (BECKMAN, Type 60 Ti) in a Beckman Optima™ LE-80K centrifuge (Beckman Coulter, Krefeld, Germany) (30 700 × g, 7 min, 4°C). Afterwards lower intermediate layer was removed with a SOFT-JECT^®^ syringe (Hartenstein, SE33) and 17G Hamilton™ needles (Fisher Scientific, 11597784). The obtained mitochondrial extracts were united in a new tube and filled with MIB up to 20 ml. After second ultracentrifugation (16 700 × g, 4°C, 12 min) mitochondria were accumulated in a fluffy pellet. In the last step, the pellet was divided into two tubes, one for RNA-isolation and one for western blot analysis. For isolation of mitochondrial RNA, the pellet was dissolved in 800 μl TRI Reagent® and mixed with 200 μl chloroform. Further steps were performed as described above. Pellet was reconstituted in 12 μl nuclease-free water.

#### Western blot

To assess quality of mitochondrial extracts, half of the obtained pellet was dissolved in 200 μl lysis buffer containing 50 mM Tris–HCl (pH 7.4,Carl Roth, 5429.5), 150 mM sodium chloride (Carl Roth, P029.3), 1% Triton™ X-100 (Carl Roth, 3051.4), 0.5% sodium deoxycholate (Sigma, 30970-25G), 0.1% sodium dodecyl sulfate (SDS, Carl Roth, CN30.2), 5 mM ethylenediaminetetraacetic acid (Carl Roth, CN06.2) and 1mM phenylmethylsulfonylfluorid (PMSF, Carl Roth, 6367.1). After incubation on ice for 45 min and centrifugation (10 000 – g, 4°C, 10 min), proteins were located in the supernatant. After protein determination (Bradford method), 20 μg protein per lane were loaded on a 12% SDS-PAGE and separated by electrophoresis. Samples were transferred onto a polyvinylidene fluoride membrane (Thermo Scientific, REF 88520), incubated for 1 h with 5% Bovine Serum Albumin (BSA) (Carl Roth, T844.3) blocking solution at RT and then incubated with primary antibodies for TOMM20 (Abcam, ab186735, 1:1000 dilution in 5% BSA) and alpha-tubulin (Abcam, ab7291, 1:5 000 dilution in 5% BSA) for 1 h at RT. After washing three times with 0.5% TBST (Tris 20 mM (Carl Roth, 5429.5), sodium chloride 150 mM, 0.5% Tween^®^ 20 (Carl Roth, 9127.1), ad 1 l water), membranes were treated with horseradish peroxide conjugated secondary antibodies. For TOMM20 an anti-rabbit antibody was used (Sigma, A0545, 1:10 000 dilution in 5% BSA) and for alpha-tubulin an anti-mouse-antibody was used (Invitrogen, 31430, 1:5000 dilution). In the last step, membranes were washed again three times with 0.5% TBST and analyzed with Amersham ECL™ Prime Western Blotting Reagent (Cytiva, RNP2236) in Fusion Pulse TS.

### *In-vitro* RNA degradation

210 μg of total RNA prepared from fresh mouse liver in a volume of 435 μl water were supplemented with 60 μl of 10x digestion buffer (250 mM NH_4A_c, pH 7.5) and degradation was started by addition of 105 μl RNAse T1/A mix (1:10 000 dilution of Thermo Fisher Scientific, EN0551). Aliquots (100 μl, corresponding to 35 μg RNA) were taken at various time points and added to an extraction mix consisting of 200 μl nuclease-free water, 150 μl phenol (Carl Roth, A980.1) and 150 μl chloroform. After agitation for 30 s, phases were separated by centrifugation (15 000 × g, 4°C, 5 min). The upper aqueous phase was re-extracted with 100 μl chloroform. The aqueous phase was supplemented with 0.5 μl glycogen and incubated with 2-propanol for 15 min at RT. After centrifugation for 30 min (15 000 × g, 4°C), the supernatant was removed and the pellet with washed with 75% ice-cold ethanol. The supernatant was discarded, and the pellet was air-dried for about 5 min. The degradation status was monitored on Agilent 4200 TapeStation system according to the manufacturers instructions. Digested RNA of each time point was separated by a 10% urea PAGE, and tRNA and RF fractions were excised as describedand processed for mass spectrometry analysis.

### post-mortem RNA degradation

Twenty mice (B6.129) were sacrificed at the same time by means of cervical dislocation. Mice were at the age of 5–6 months and in total 10 male and 10 female mice were used. Whole brain and liver pieces of the left lobe (∼200 mg) were dissected after different storage times (0, 1, 8, 24 h) at RT, to monitor *in-vivo* degradation post-mortem. After dissection tissue was shock frozen in liquid nitrogen and stored in −80°C freezer until isolation of RNA the next day. Thereby two male and two female mice were chosen for every time point. In addition, two female and two male mice were dissected and RNA-Isolation was performed immediately afterwards without freezing to specify the effect of freezing. RNA-Isolation, purification of tRNA and RF-fractions, and LC−MS analysis was done as described above.

### Probing differences between brain tissues in mice

Cohort 1, from Mainz, Germany: Female and male C57BL/6JRj mice were sacrificed at the age of 12 weeks on the same day. Hippocampus, cortex and cerebellum were dissected manually. For the sake of sufficient RNA input amount, brain areas of two individuals were pooled resulting in four biological replicates. All biological replicates were submitted to a 10% urea PAGE and tRNA- and RF-RNA fractions were isolated and analyzed for their modification content as described above. Biological samples with high levels of ribosomal markers Am and m^6,6^A in tRNA fractions, were considered degraded and excluded from further analysis according to the rationale developed in the results and discussion section.

## RESULTS

At the onset of our investigations, we posited that modifications, properly quantified in cellular RNA, should be informative of several features of the state of a tissue or cell culture, potentially including aspects of cell death, protein synthesis capacity, and mitochondrial biomass or activity (39). As an equally important second aspect, we anticipated finding modifications from degradation fragments originating from large RNAs such as rRNAs, mixed with small RNAs. We felt that a quantitative assessment of this phenomenon would be of high importance to the community, given scattered reports on the detection of modifications in small RNAs that conform to this prediction (22). As a first aspect, we focused on the integrity status of preparations of total RNA in the perspective of intracellular and extracellular decay. We consequently investigated RNA fractions of defined size and searched for characteristic modifications that can be used to assess its degradation status.

Based on literature on the occurrence of modifications in different RNA species (18,40), we postulated the following experimentally verifiable hypotheses. These are: (i) different RNA classes should be characterized by signature modifications and (ii) the latter should be enriched or even uniquely present in RNA fractions of different sizes. Furthermore, (iii) progressive degradation should shift the distribution of signature modifications towards fractions of sizes smaller than that of the original intact species, such that (iv) the presence and quantity of certain marker modifications in small RNA fractions would be a measure of the overall degradation status of the total RNA sample. Especially in hypothesis (iv), an obvious objective was the identification and validation of such marker modifications.

### Signature modifications are characteristic for RNA preparations of different size

The substantial body of knowledge on the modification content of eukaryotic tRNA, rRNA and mRNA in the literature firmly establishes that, despite similarities, each species, tissue, and even physiological state features a specifically adapted epitranscriptome (17,41,42). For our purpose, this necessitated the initial characterization of a starting point. We therefore used fresh mouse liver to prepare samples of tRNA, rRNA and polyA-RNA, the latter consisting mostly of mRNA and presumably containing an as yet ill-defined fraction of polyadenylated ncRNA (43–45). We will consider the case of tRNA fragments and other RNAs in the size range around 35 nucleotides in due course.

**Figure 1.**
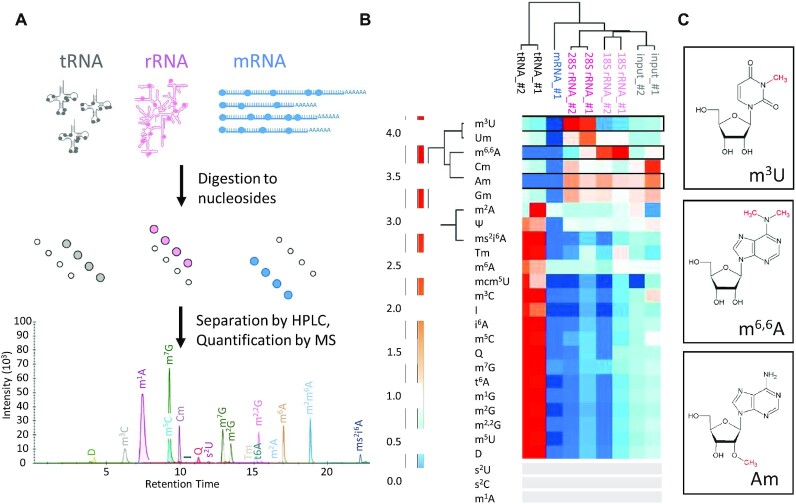
Distinct modification content of major cellular RNA classes. (**A**) Workflow of RNA modification measurement. Nucleosides resulting from complete digest of isolated RNA samples were separated by HPLC and quantified by MS/MS. For technical reasons, the presented chromatogram only illustrates 19 out of 27 actually measured modifications (**B**) Heatmap comparing modification patterns of tRNA (black), mRNA (blue), input (total RNA, grey), 28S rRNA (pink) and 18S rRNA (rose) of mouse liver tissue. *n* = 3 technical replicates. Two biological replicates (#1&#2) were prepared, except for mRNA; for each RNA type, 3 technical replicates per biological sample were measured. (**C)** Chemical structure of the candidate modifications for ribosomal markers: m^6,6^A from 18S rRNA, m^3^U from 28S rRNA and Am from both rRNA types.

Using a stable isotope labelled internal standard (SILIS) (38) the modification content of these RNA fractions was quantified for a total of 27 different modifications as illustrated in Figure 1A. To this end, RNA preparations were digested to mononucleosides and supplemented with SILIS as detailed in the material and methods section. Quantitative results are given in Table 2, and Figure 1B, shows a heatmap of the distribution of modifications among the different classes of RNA. The hierarchical clustering on the x-axis of rRNAs, tRNAs, mRNA and the input RNA (Figure 1B) cleanly groups each class of RNA and separates it from the respective others, experimentally validating our hypothesis (i) – *vide supra*. Total RNA (labelled ‘input’) clusters with the rRNA, plausibly as a consequence of the large mass contribution (>80%) of rRNA to cellular RNA populations.

**Table 2.** Absolute quantification of RNA modifications. The numbers correspond to the modification content of species in Figure 1B. Only those 17 modifications for which SILIS and external calibration standards were available could be quantified in abolsute terms, and for lack of a defined sequence, results arenormalized to the UV signal of adenosine [%]. bdl = below detection limit. * = quantification of this peak has exceeded calibration range.

	tRNA #2	tRNA #1	mRNA #1	28S rRNA #2	28S rRNA #1	18S rRNA #2	18S rRNA #1	Input #2	Input #1
m^3^U	0.044	0.035	bdl	0.273	0.214	0.015	0.024	0.050	0.055
Um	2.349	1.913	bdl	3.102	5.258	2.600	2.603	1.587	1.594
m^6,6^A	0.013	0.010	0.006	0.154	0.490	0.950	1.469	0.278	0.451
Cm	0.523	0.426	0.017	0.887	0.594	0.585	0.444	0.684	1.533
Am	0.034	0.024	0.043	2.754	2.070	2.555	2.017	1.915	2.949^a^
Gm	1.335	1.066	0.069	2.949	1.829	1.907	1.539	1.876	2.328^a^
m^6^A	0.649	0.498	0.229	0.230	0.240	0.173	0.319	0.306	0.304
m^3^C	0.602	0.466	2.0E-04	0.006	0.032	0.006	0.057	0.090	0.202
I	0.884	0.670	bdl	bdl	0.057	bdl	0.096	0.217	0.167
i^6^A	0.204	0.149	1.5E-04	0.002	0.011	0.002	0.020	0.031	0.036
m^5^C	7.484	6.279	0.011	0.354	0.632	0.114	0.783	1.355	*2.273
m^7^G	4.664	4.070	0.149	0.097	0.349	0.358	0.666	0.880	1.026
t^6^A	0.703	0.590	bdl	0.008	0.046	0.008	0.075	0.121	0.138
m^1^G	2.975	2.500	0.001	0.032	0.170	0.034	0.297	0.486	0.565
m^2^G	6.193	5.077	bdl	0.059	0.328	0.054	0.583	0.987	1.135
m^2,2^G	2.293	1.890	4.9E-04	0.020	0.125	0.020	0.222	0.360	0.454
m^5^U	3.083	2.529	bdl	0.041	0.195	0.038	0.303	0.482	0.609

bdl = below detection limit.

^a^Quantification of this peak has exceeded calibration range.

The characteristic patterns of modification content in different RNA populations apparent from the heatmap in Figure 1B clearly suggest, that one may indeed draw conclusions as to the composition of an RNA sample from its modification content, and to potentially identify modifications-contributions of major RNA classes in the process. Unsupervised clustering on the Y-axis neatly groups characteristic ribosomal modification m^3^U, Nm and m^6,6^A on one hand, and typical tRNA modifications on the other hand.

Among the most characteristic modifications is m^6,6^A, which occurs almost exclusively in the small ribosomal subunit 18S rRNA. m^3^U as counterpart, is typical for the large subunit 28S rRNA (18). Ribose methylations Nm (Um, Cm, Gm, Am) occur in rRNA at high frequency, but most are present in tRNA as well. As the exception, Am is not known to occur in tRNA and the corresponding signal is indeed essentially absent from the tRNA fraction, as well as from the mRNA, and might therefore be a marker for rRNA from either subunit. The remaining three Nm occur in both, tRNA and rRNA fractions. Consequently, with an eye to our hypothesis (ii), Gm, Cm and Um are unlikely to qualify as good marker modifications, whereas we retain m^6,6^A and m^3^U (Figure 1C) as candidates for their respective subunit rRNA, and Am as a potential generic marker for rRNA.

The tRNA fraction contains a number of characteristic ‘classical’ modifications, such as e.g. m^5^U, whose sole known occurrence outside eukaryotic tRNA is in the large subunit of mitochondrial rRNA (46,47) (), as well as Tm, D and ms^2^i^6^A.

Of note, the nucleolytic enzymes and phosphatases used in this protocol will liberate m^7^G from corresponding cap structures and cause a corresponding signal e.g. in polyA-RNA preparations, as verified using enzymatically capped, *in vitro* transcripts of poly-A containing mRNA. In keeping with this, the polyA fraction contains m^6^A, m^7^G (Table 2) in addition to traces of other modifications (including m^6,6^A), but all of them in lesser amounts than in other RNA fractions, as can be seen in the heatmap of Figure 1B. Altogether, the heatmap highlights that among the quantified nucleoside modifications, none are abundant enough in mRNA to become a characteristic ‘mRNA marker’, not even the much observed m^6^A. The presence of m^6,6^A corroborates small amounts of rRNA contaminations, which were already anticipated from a peak of residual rRNA in the electropherogram of the purified polyA fraction (Supplementary Figure S1). This type of contamination is highly reproducible in samples from various origins that were investigated in other settings in this lab (not shown). We surmise that this represents a polyadenylated fraction of rRNA, potentially targeted for degradation by the exosome (2).

### *In vitro* degradation can be monitored via a redistribution of signature modifications

In addition to the large quantities of potential marker modifications in rRNA itself, we noticed that traces of typical rRNA modifications were also present in the tRNA fraction. In particular, we found small amounts of Am, m^6,6^A and m^3^U, which have thus far not been identified in mammalian cytosolic tRNA (Table 1). We hypothesized that these might stem from degradation events having occurred either in the living cell, in dead, necrotic or apoptotic cells, or during the RNA isolation procedure.

**Figure 2.**
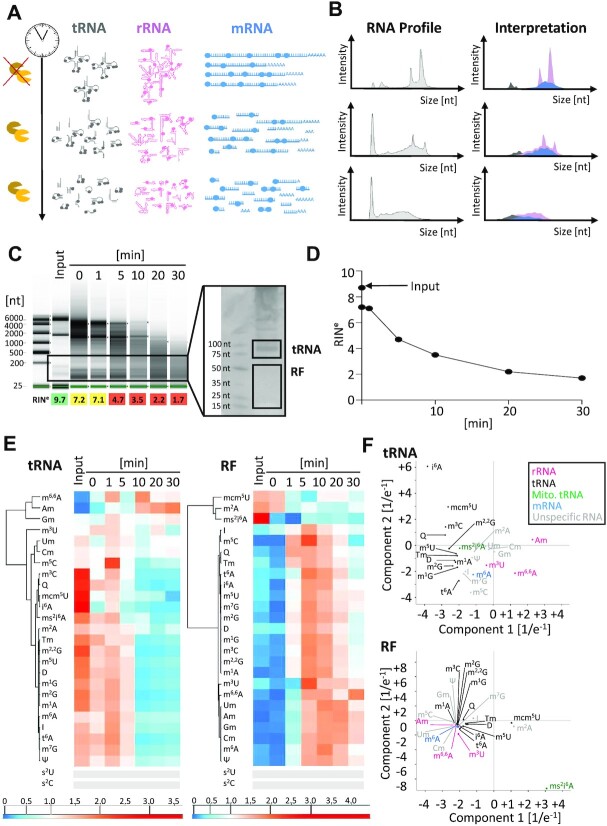
*In vitro* degradation of total RNA from mouse liver tissue. (**A**) Schematic representation of RNase-induced time dependent degradation process of the three major RNA classes: tRNA (grey), rRNA (pink) and mRNA (blue) are fragmented over time. (**B**) Corresponding RNA profiles as obtained by capillary electrophoresis (CE) are shown in grey. The right panel visualizes an interpretation of the redistribution of RNA species and their modifications in the same colors as in (A). (**C**) CE profiles of total RNA after in vitro degradation at indicated time points. A preparative 10% urea PAGE was used for isolation of RNAs of tRNA or RF size (right panel). (**D**) Quantification of the decay kinetics as plot of RIN^e^ values over time. (**E**) Heatmaps of tRNA and RF fractions clustered for modified nucleosides. Each row was normalized by its mean. *n* = 2 technical replicates for RF-fraction, *n* = 3 technical replicates for tRNA. (**F**) Principal component analyses of tRNA and RF samples from (**E**) modifications with signals below 5× S/N across all samples are represented by grey bars.

To simulate the latter, degradation events of total RNA were re-enacted in an *in vitro* time course of degradation *via* treatment with RNases, as shown in Figure 2A, for verification of the distributions of RNA size and modifications upon degradation. The progressive degradation of a sample of total RNA was effectively mirrored in the corresponding capillary electrophoresis (CE) profiles (Figure 2B−D). The right column in Figure 2B shows a graphic representation of our hypothesis (iii)—*vide supra—*that anticipates features of the progressive degradation, broken down into color-coded partially degraded subpopulations of tRNA, mRNA and rRNA. Accordingly, we expectedf an increasing occurrence of signature modifications of the larger RNA, in particular of rRNA, in fractions of smaller RNAs.

For experimental verification, RNA samples from a degradation experiment were size fractionated by PAGE, excised from gel slabs, eluted, precipitated, and analysed for their modification content by LC–MS as before (Supplementary Figure S2). The results are shown as heatmaps (Figure 2E) for RNAs of tRNA size and for smaller fragments, labelled ‘RF’. Of note, in the tRNA fraction, the previous marker characteristics hold, e.g. for rRNA modifications, in which m^6,6^A clusters with the various Nm and neighbour m^3^U. This is also reflected, to a degree, in a principle component analysis (PCA) which is shown in Figure 2F (upper panel). A PCA can be useful to identify coherent behaviour of entities (here: modifications) in different parameters. Here, the PCA shows, that m^6,6^A and Am truly stand characteristically separated from the rest, presumably because they are contained in rRNA fragments of similar size, undergoing similar degradation events. Their covariation effectively validates these two modifications as small subunit rRNA markers according to our hypothesis (ii). Among the smaller RNAs in the RF fraction, however, there is no more coherent behaviour among the rRNA modifications; a plausible explanation would be that they enter the tRNA fraction in fragments of similar size and metabolic stability, while these properties would diverge upon further degradation.

In summary, the progressive degradation causes two opposing trends, namely an enrichment of rRNA modifications in the tRNA fraction, and in the same fraction, a dilution of tRNA modifications. The latter simultaneously become enriched in the RF fraction, effectively validating out hypothesis (iii).

### ms^2^i^6^A is a mitochondrial marker

Of some interest is the detection of ms^2^i^6^A, a lipophilic tRNA hypermodification of prokaryotic origin, that is known to occur in mammalian mitochondria, but not in the cytosol or nucleus (47–49). In the PC analyses of modifications of the tRNA fraction in Figure 2F, this modification clusters with other known tRNA modifications, but in the RF fraction it exhibits clearly distinct characteristics, potentially due to mitochondria-specific tRNA degradation behaviour. To validate its use as mitochondrial marker, we purified mitochondria from liver and brain using an extended protocol, which included in particular a two-step percoll gradient (Supplementary Figure S3). Cytosolic and mitochondrial protein markers were traced by western blot: the resulting material contained the mitochondrial marker TOMM20, a component of the outer mitochondrial membrane transport system, but no more cytosolic α-tubulin protein. Supplementary Figure S3 shows a heatmap of RNA preparations from the parent tissues brain and liver, as well as of the respective mitochondrial preparations. The mitochondrial preparations are clearly distinct from the total RNA of ‘input’ material, both for brain and liver preparations. The brain mitochondrial preparations, in turn, are clearly distinct from the liver mitochondrial preparations, and the difference is based on an increase of tRNA modifications and a decrease of rRNA marker modifications in brain versus liver. In addition to detecting a co-purification of ms^2^i^6^A with mitochondrial RNA, which effectively validates its mitochondrial marker status (hypothesis (i)), brain mitochondria contain high amounts of queuosine, a wobble modification directly involved in the mRNA decoding process.

### the RNA morgue: application of signature modifications to a post-physiological setting

Having characterized *in vitro* degradation as a potential source of rRNA fragments in small RNA preparations, we turned to more physiologically plausible origins of degraded rRNA, such as necrotic or apoptotic processes. These cause a certain amount of dead cells among cells in tissue as well as in tissue culture, and the resulting dead cells can reasonably be expected to contribute RNA to total RNA preparations. Given that degradation processes commence during cell death already, it can furthermore be assumed to follow at least partially predefined pathways. Consequently, the resulting degradation patterns would likely be different from that caused by the artificial *in vitro* settings above.

**Figure 3.**
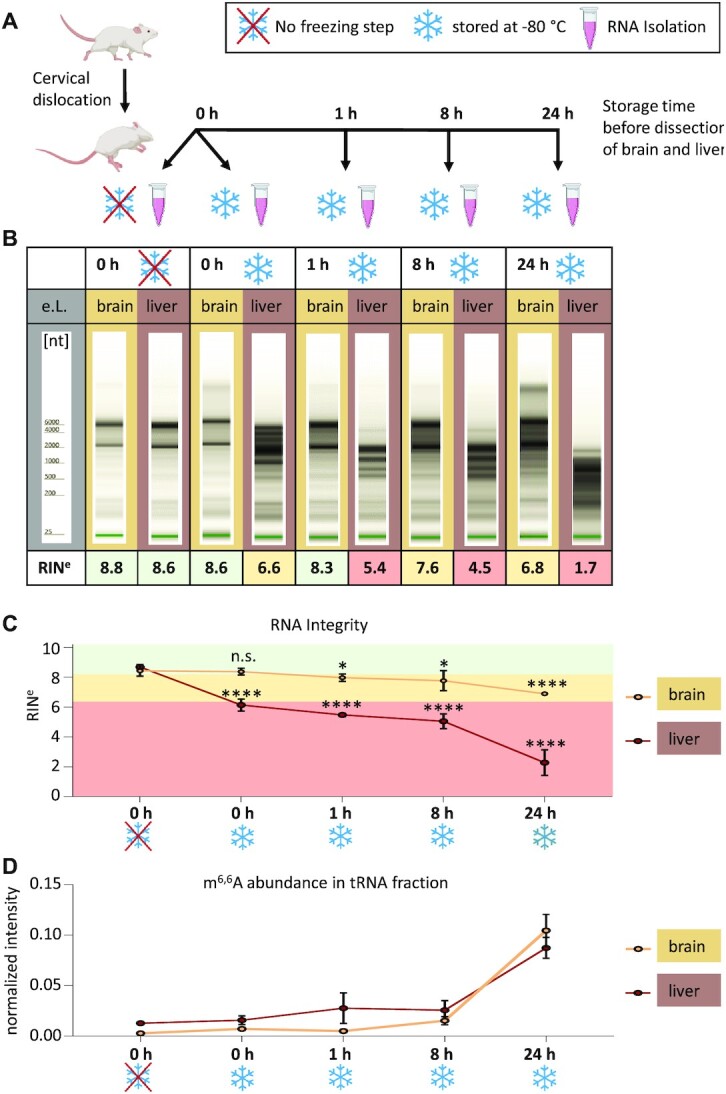
*In cellulo* degradation of total RNA after organismal death. (**A**) Workflow: mice were sacrificed by cervical dislocation. RNA from liver and brain tissue was isolated at different time points *post mortem*. Additionally, the effect of shock freezing was assessed from RNA isolated immediately after dissection. (**B**) Representative TapeStation Profiles of total RNA from brain (light brown) and liver (dark brown), respectively, at the time points indicated in A. (**C**) RNA integrity (RIN^e^) values of liver and brain RNA plotted over time (mean ± SD, *n* = 4 biological replicates, n.s. = not significant, *P< 0.05, ****P< 0.0001, unpaired *t*-test, compared to 0 h without freezing). (**D**) Relative Quantification of the rRNA marker m^6,6^A by LC–MS/MS shows elevated m^6,6^A levels in tRNA fraction of liver and brain RNA in a time-depentend manner. Mean ± SD, *n* = 4 biological replicates.

**Figure 4.**
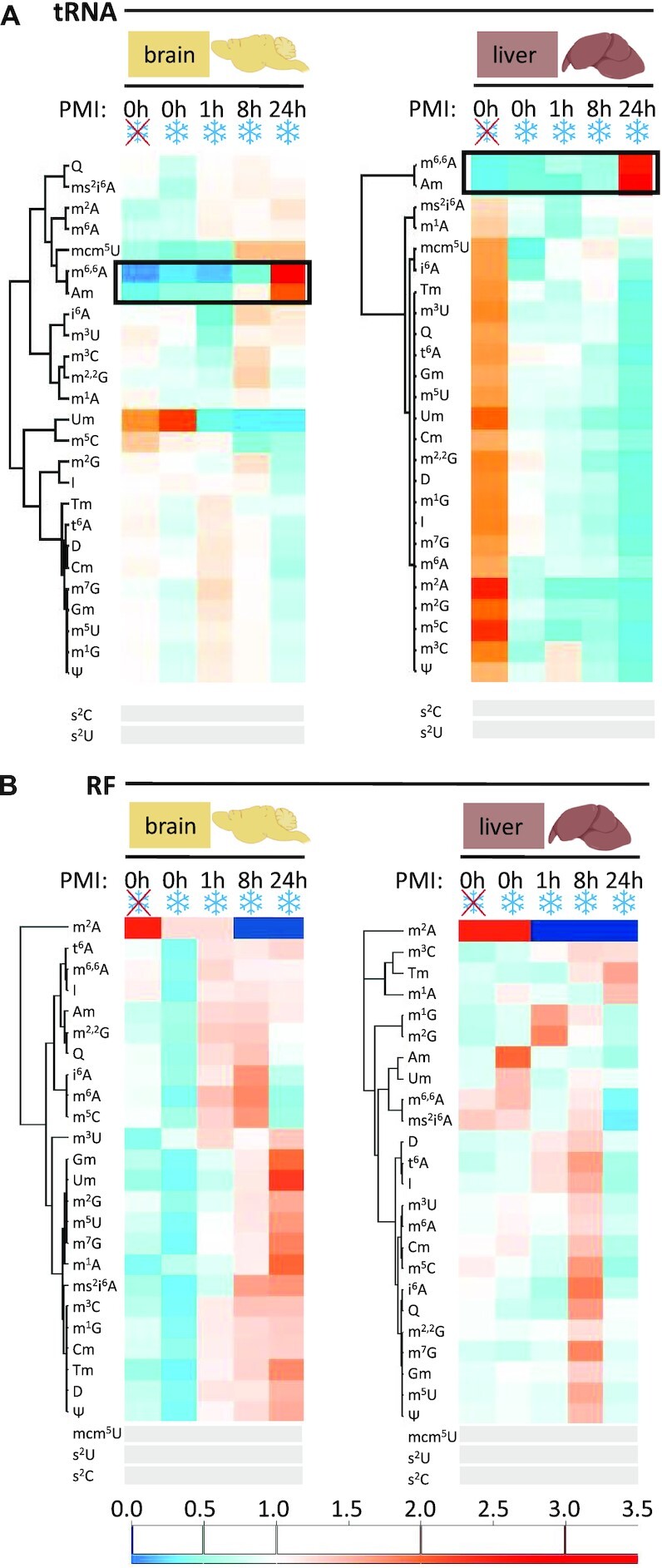
Comparison of modification profiles in tRNA and RF upon *in cellulo* degradation post-mortem. (**A**) Heatmaps represent modification content of tRNA-fractions from mouse brain and liver tissue isolated at different time points post mortem and in comparison with unfrozen samples. Each row was normalized by its mean. *n* = 4 biological replicates. (**B**) At different time points *post mortem* and in comparison with unfrozen samples. Modifications with signals below 5x S/N across all samples are represented by grey bars.

Here, organismal death offers an advantageous setting, since it causes a synchronized onset of cell death in tissues. To gauge the influence of tissue type, and the differentiated proteome associated with it, we compared RNA degradation *in cellulo* between brain and liver tissue after organismal death. Mouse brain and liver were removed at defined time points of 0, 1, 8 and 24 h after death (Figure 3A), and RNA was isolated and analyzed for its degradation (Figure 3B, C), as well as for modification profiles of its tRNA (Figures 3D,4, S4) and RF fractions (Figures 4, S4).

First, two different isolation procedures were tested to gauge the influence of flash freezing and thawing on degradation. In one case, tissue was flash frozen in liquid nitrogen and later minced in RNA isolation solution *after* thawing. Alternatively, the issue was directly minced and treated with RNA isolation solution. As can be seen in Figure 3B, the thawing process significantly contributed to RNA degradation in liver, but not in brain (brain: n.s., liver: ****P < 0.0001). Degradation in liver was fast progressing in comparison to brain, where even after 24 h, total RNA was still mostly intact. The accelerated degradation in liver relative to brain is easily appreciated in a plot of integrity values vs. time (Figure 3C). A comparative analysis of the modification content of the tRNA fraction, respectively, revealed an enrichment of the rRNA maker modification m^6,6^A with increasing degradation, becoming visible as early as one hour *post mortem* in liver, and only after 8 hours in brain (Figure 3D). At 24 h, both tissues show an even higher m^6,6^A content. This data establishes ribosomal m^6,6^A as a useful marker for the assessment of an RNA preparations’ degradation status, and thus effectively validates our hypothesis (iv)—*vide supra*.

A comparative kinetic characterization of the tRNA and RF fractions *via* heatmaps, as shown in Figure 4, revealed divergent behavior for various modifications as a function of tissue and provenance in terms of RNA species. The degradation in liver tissue did recapitulate the *in vitro* degradation (Figure 2) for the most part: rRNA marker modifications (m^6,6^A, Am) were increasing in the tRNA fraction with increasing degradation, while tRNA modifications diminished (Figure 4A, right panel). Again, the RF fraction does not follow a clear pattern in either liver or brain, and also rRNA derived modifications are present in the RF fraction, but are not enriched with progressive degradation (compare Figure 4B and Figure 2). Furthermore, tRNA specific modifications constantly increase in the brain RF fraction but start to fade away in the liver RF fraction already after 24 h, in conjunction with the higher overall degradation rate in liver. Time courses of selected modifications are shown in Supplementary Figure S4.

Not surprisingly, known tRNA modifications group together in the hierarchical clustering of the tRNA fractions from either tissue. Interestingly, ribose methylated adenosine Am continues to cluster with m^6,6^A, confirming its prevalent occurrence as rRNA modification, in line with its behavior in the previous experiments (Figures 1 and 2). Other known rRNA modifications such as Gm and Um (Figure 1), however, diverge in their clustering, presumably due to their simultaneous occurrence in e.g. tRNA. Also of interest, m^3^U, which is so far only known to occur in the large subunit rRNA, does not replicate the behavior of small subunit rRNA markers m^6,6^A and Am, presumably undergoing a different degradation pathway.

The above data outlines, that not only modification patterns of intact RNA, but also the degradation kinetics, as observed by modification patterns, retain tissue specific character. Clearly, great care must be taken in the preparation and interpretation of RNA data from liver in general. By the same token, the data effectively validates the RNA preparations from brain, immediately postmortem and without freezing, as pristine, i.e. free of contaminations caused by degradation *in vitro* or *in cellulo*. This allowed us to continue our investigations of RNA modifications in brain tissue in order to gauge its full potential in characterization its metabolic state.

### Distinct tRNA modification profiles in different brain tissues

**Figure 5.**
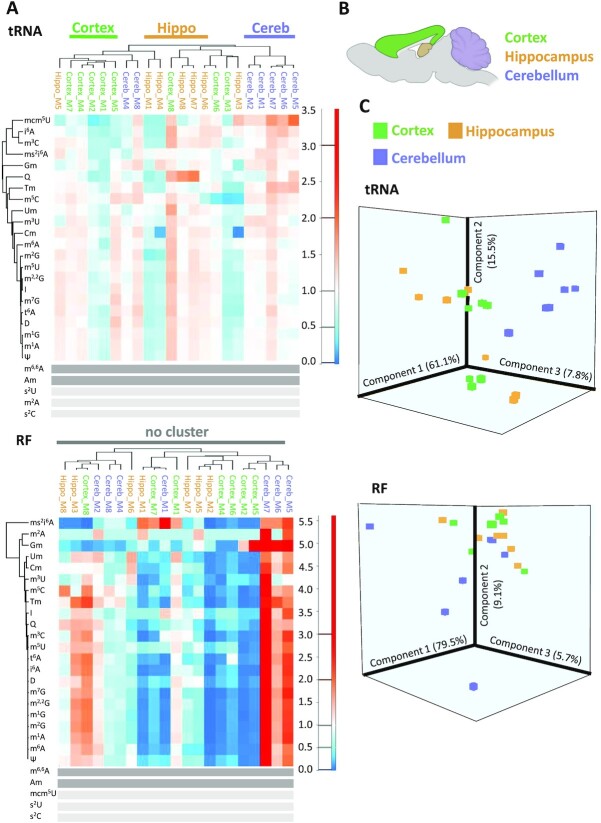
Comparison of different mouse brain tissues (cohort 1). (**A**) Heatmap displaying tRNA and RF modification content of different brain areas. From *n* = 16 mice, the material of two mice were pooled, resulting in 8 biological replicates. (**B**) Sketch of localization of cortex, hippocampus and cerebellum in a mouse brain sagittal cut. (**C**) Principal component analysis of brain area samples from (B). rRNA modifications in dark grey were omitted from the analysis. Modifications with signals below 5× S/N across all samples are represented by light grey bars.

Given the very clear distinction of modification profiles between brain and liver tissue, we investigated, whether it was possible to achieve a distinction even among tissues that are more closely related. Because our previous investigations had shown that cellular RNA remains undegraded for extended periods of time, we isolated RNA from three different brain tissues, namely hippocampus, cerebellum and cortex. Given the multitude of modifications found in cellular tRNA and tRNA derived fragments, we analyzed the corresponding modifications across a cohort #1 of 7 samples (each of the seven samples contained brain material pooled from two animals). Figure 5 shows the corresponding heatmap, where a comparison of tRNA modification content (deliberately omitting the established rRNA markers Am and m^6,6^A) allows a clear distinction of all three tissues via unsupervised hierarchical clustering. As before, there is also a distinction of tRNAs versus RNA fragments, but only the tRNA profiles are informative with respect to tissue distinction. Of interest, tissue distinction within the tRNA fraction is also evident in a 3D PCA analysis (Figure 5C). For verification, we analyzed a different mouse population (cohort #2), which was grown in a different lab (here: in Heidelberg). The cerebellum, cortex, and hippocampus tissues were separated by a different person, who also performed tRNA extraction. Modification analysis was again conducted according to the same protocol and on the same machine (in Mainz). The results, which are shown in Supplementary Figure S5 also show a tissue specific segregation in unsupervised clustering although less pronounced, and the corresponding PCA separates the three different tissues as well.

## DISCUSSION

Post mortem RNA degradation, though a plausible source of RNA artefacts, has remained largely ignored as a source of fragmented small RNAs in both, RNAseq and LC-MS based investigations in epitranscriptomics. By analysing decay processes after organismal death, we here established a baseline to gauge the minimum occurrence of rRNA degradation encountered in RNA preparations from fresh tissue. A number of important conclusions flow from these results, pertaining to the identification and validated use of marker modifications, their size distribution upon RNA degradation, and the application to RNA from different tissues.

### Validation of marker modifications

Degradation resulted in a clear increase of the co-varying rRNA modifications m^6,6^A and Am in the tRNA fraction, but not in the RF fraction, *in vitro* as well as *in cellulo*, and in liver as well as in brain. A plausible explanation here must assume that this fraction is, from the very onset, in a steady-state, where the amount of e.g. m^6,6^A/Am containing fragments being degraded equals that of newly generated fragments. In contrast, the RNA fraction of tRNA size does accumulate rRNA fragments in all settings investigated, meaning that e.g. the m^6,6^A/Am content in the tRNA fraction can serve as a marker of the overall degradation state of a given sample.

In some contrast, tRNA modifications decay in the tRNA fraction upon *in vitro* degradation and *in cellulo* degradation in liver, but not in brain, the latter being remarkably inert to degradation altogether. Of note tRNA degradation alone can not *per se* explain this observation, since there must be an increase of RNA fragments of tRNA size that do not contain tRNA modifications. Since a massive surge in *de novo* transcription of tRNA is implausible, the decrease of tRNA modification content in an RNA fraction of tRNA size is best explained by a dilution of this fraction with rRNA fragments of corresponding size, which do not carry the respective tRNA marker modifications. Initially, the decrease of typical tRNA modifications in the tRNA fraction is paralleled by an increase in the FR fraction (Figure 2E*in vitro* and Figure 4B in liver), which however, is transient, as these modifications go through a maximum and are decreased again at high degradation state (30 min *in vitro* and 24 h in liver). In brain, degradation is very slow and especially changes in tRNA modifications are much less pronounced in the tRNA fraction, whereas they do increase in the RF fraction at later time points (Figure 4), re-enacting the processes in liver at a much slower time scale.

These observations raise the intriguing possibility of modelling the ‘flux’ of tRNA copy number, modification stoichiometry, and fragmentation status. Such a model would have to employ advanced methods of tRNA isoacceptor quantification as well as sequence specific modification mapping and quantification of modification stoichiometry. Our tentative forays into this area have clearly indicated the need for many more data points to model that many parameters.

### Implications for RNA preparations from different tissues

A most sobering observation was the fast decay of RNA integrity in liver already during a simple flash-freeze and thaw procedure (Figure 3B), plausibly a consequence of the high metabolic activity of liver tissue. The associated drastic changes in modification content (Figure 4A) illustrate the problem of contaminating rRNA fragments in fractions of smaller RNAs. Future studies will have to combine RNAseq and LC–MS to gain a quantitative understanding of the physiological relevance of RNAs contained in such isolates. Importantly, future analyses of transcriptomes and epitranscriptomes will have to revise isolation procedures for each different tissue type, at the very least for the more metabolically active tissues such as liver. Indeed, our observations may be taken as an incentive to revisit certain such investigations.

On the other hand, the pronounced stability of RNA preparations from brain opens up avenues for assessment of its modification content in different tissue types, as here exemplified by tRNA modification patterns that show similarities among isolates of cerebellum, hippocampus, and cortex (Figures 5 and S5). Of note, these differences do not rely on the abundance of single modifications, which by themselves could be assessed via a simple *t*-test, but rather on differences of groups of tRNA modifications, made visible by PCA or unsupervised clustering. While the clustering did show significant similarities, it was imperfect (Figure 5B), but the PCA illustrated similarities quite well. Of note, the RF fraction serves as an effective negative control, where similarities are not seen in either type of analysis. After concluding the experiments shown in Figure 5 in Mainz, we contacted a partner lab in Heidelberg, to exclude artefacts as much as possible. Thus, tRNA preparations from the same three brain tissues were analysed, that had been prepared by a different person at a different site. Again, the unsupervised clustering of samples showed a tendency, despite imperfect segregation, and again, the PCA illustrated similarities.

Together, these results prompt us to conclude that, based on the prior elaboration of degradation status *via* modification markers, our analysis demonstrates variations in the tRNA epitranscriptome of different brain tissues. Pending a similar assessment of e.g. rRNA modifications, we predict that similar analysis will allow a distinction between many more different tissues, paving the way to a fast characterization of a significant part of the respective epitranscriptomes.

### The perception of small RNAs is coming full circle

As outlined, the early days of small RNA research have seen a challenge of the ‘debris’ at the bottom of PAGE gels (9–12). Numerous breakthrough discoveries of biological functions of RNAs from this fraction have, over decades, driven home the notion, that all RNAs in this fraction serve a very defined biological function in a corresponding pathway. A striking recent example pertains to the m^6,6^A rRNA marker modification, whose whereabouts we have traced in here in detail. The presence of m^6,6^A in was detected in ‘small’ RNAs by LC–MS (50) and its presence was dependent on the known rRNA MTase Dim1. And while this work was technically well executed, the most straightforward interpretation, namely degradation, was downplayed in favor of a hypothesis of m^6,6^A-containing small RNAs with biological function, whose sequences were, however, not analyzed. Here, our findings point out an important, but by now overlooked fact: there are indeed debris in the small RNA fraction, and some of them are just that: degradation products on their way to recycling.

## ABBREVIATIONS

A selected list of abbreviations is available in the Supplementary Data.

## Supplementary Material

gkab1150_Supplemental_File
