# Supplementary material for: RNA marker modifications reveal the necessity for rigorous preparation protocols to avoid artifacts in epitranscriptomic analysis
Source: Nucleic Acids Res. 2021 Dec 1;50(8):4201–15. doi: 10.1093/nar/gkab1150 (PMC9071408; doi:10.1093/nar/gkab1150)
Supplement: gkab1150_Supplemental_File [file gkab1150_supplemental_file.pdf]

Supplemental Material to

RNA marker modifications reveal the necessity for rigorous preparation protocols to avoid artifacts in epitranscriptomic analysis

Florian Richter[1,†], Johanna E. Plehn[1,†], Larissa Bessler[1], Jasmin Hertler[1], Marko Jörg[1], Cansu Cirzi[2,3], Francesca Tuorto[4,5], Kristina Friedland [1,*], and Mark Helm[1, *]

Page s2-3	Abbreviation list
Page s4	Supplementary Figure S1. Capillary electrophoresis of polyA RNA
Page s5	Supplementary Figure S2. Preparative isolation of *in vitro* degraded RNA.
Page s6	Supplementary figure S3. Enrichment of mitochondria tracked by modification profiles
Page s7	Supplementary figure S4. Relative abundance of specific RNA modifications over time in tRNA & RF fractions during the RNA morgue experiment.
Page s8	Supplementary figure S5. Comparison of different mouse brain tissues (cohort 2).

ABBREVIATIONS

RNA	Ribonucleic acid
tRNA	Transfer RNA
rRNA	Ribosomal RNA
mRNA	Messenger RNA
RF	RNA Fragments
PAGE	Polyacrylamide gel electrophoresis
UV/VIS	Ultraviolet/ Visible spectroscopy
RIN^e	Electronical RNA integrity number
h	hours
d	days
RT	Room Temperature
L	liter
ml	Milliliter
µl	Microliter
µg	Microgram
PCA	Principal component analysis
SILIS	Stable isotope labelled internal standard
SED	Standard Error of Difference
TBE	Tris-Borat-EDTA buffer
mA	Miliampere
V	Volt
M	Molar concentration
FastAP™	Fast alkaline phosphatase
U	(enzymatic) units
HPLC	High pressure liquid chromatography
MS	Mass spectrometry
ACN	Acetonitrile
MWD	Multiple wavelength detector
AUC	Area under the curve
MPI	Max Planck Institute
PBS	Phosphate-buffered saline
MIB	Mitochondrial isolation Buffer
TOMM20	Translocase of outer mitochondrial membrane 20
PMSF	Phenylmethylsulfonylfluoride
TBST	Tris-buffered saline with Tween®
polyA	Polyadenylation
CE	Capillary electrophoresis
BSA	Bovine Serum Albumin
n.s.	Not significant
p-value	Probability value

RNA Modifications:

Am	2’-O-methyladenosine
Cm	2’-O-methylcytidine
D	Dihydrouridine
Gm	2’-O-methylguanosine
I	Inosine
i^6A	N6-isopentenyladenosine
m^1A	1-methyladenosine
m^2A	2-methyladenosine
m^6A	N6-methyladenosine
$m^{6,6}A$	N6,N6-dimethyladenosine
m^3C	3-methylcytidine
m^5C	5-methylcytidine
mcm^5U	5-methoxycarbonylmethyluridine
m^1G	1-methylguanosine
m^2G	N2-methylguanosine
$m^{2,2}G$	N2,N2-dimethylguanosine
m^7G	7-methylguanosine
ms^2i^6A	2-methylthio-N6-isopentenyladenosine
m^3U	3-methyluridine
m^5U	5-methyluridine
s^2C	2-thiocytidine
s^2U	2-thiouridine
t^6A	N6-threonylcarbamoyladenosine
Tm	2’-O-methylthymidine
Um	2’-O-methyluridine
Q	Queuosine
Ψ	Pseudouridine

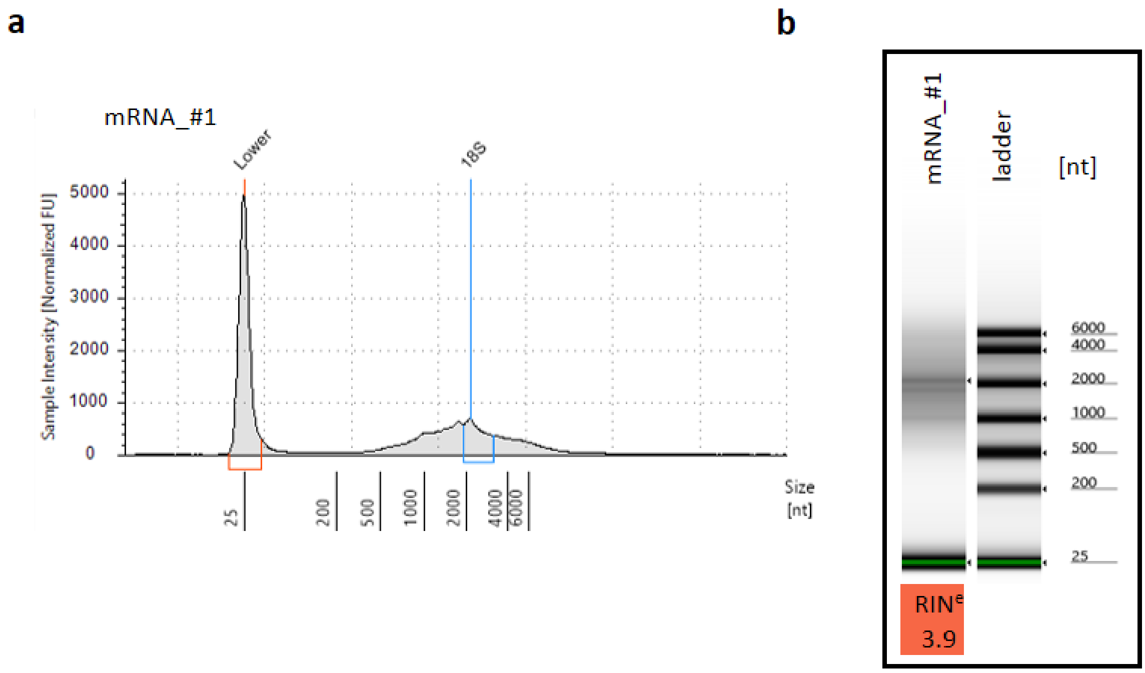

Supplementary Figure S1. Capillary electrophoresis of polyA RNA. The RNA was isolated according to Werner *et al.,* using double hybridization to oligo-dT. The resulting RNA still contains peaks in the typical size range of rRNA. (a) Size distribution of mRNA#1 using capillary electrophoresis. (b) CE profile of mRNA#1 compared to the standard ladder.

Werner, S., Galliot, A., Pichot, F., Kemmer, T., Marchand, V., Sednev, M.V., Lence, T., Roignant, J.-Y., König, J., Höbartner, C., Motorin, Y., Hildebrandt, A., **Helm, M.**, NOseq: amplicon sequencing evaluation method for RNA m6A sites after chemical deamination, (**2021**), *Nucleic Acids Res.*, Feb. 26, 49(4):e23,

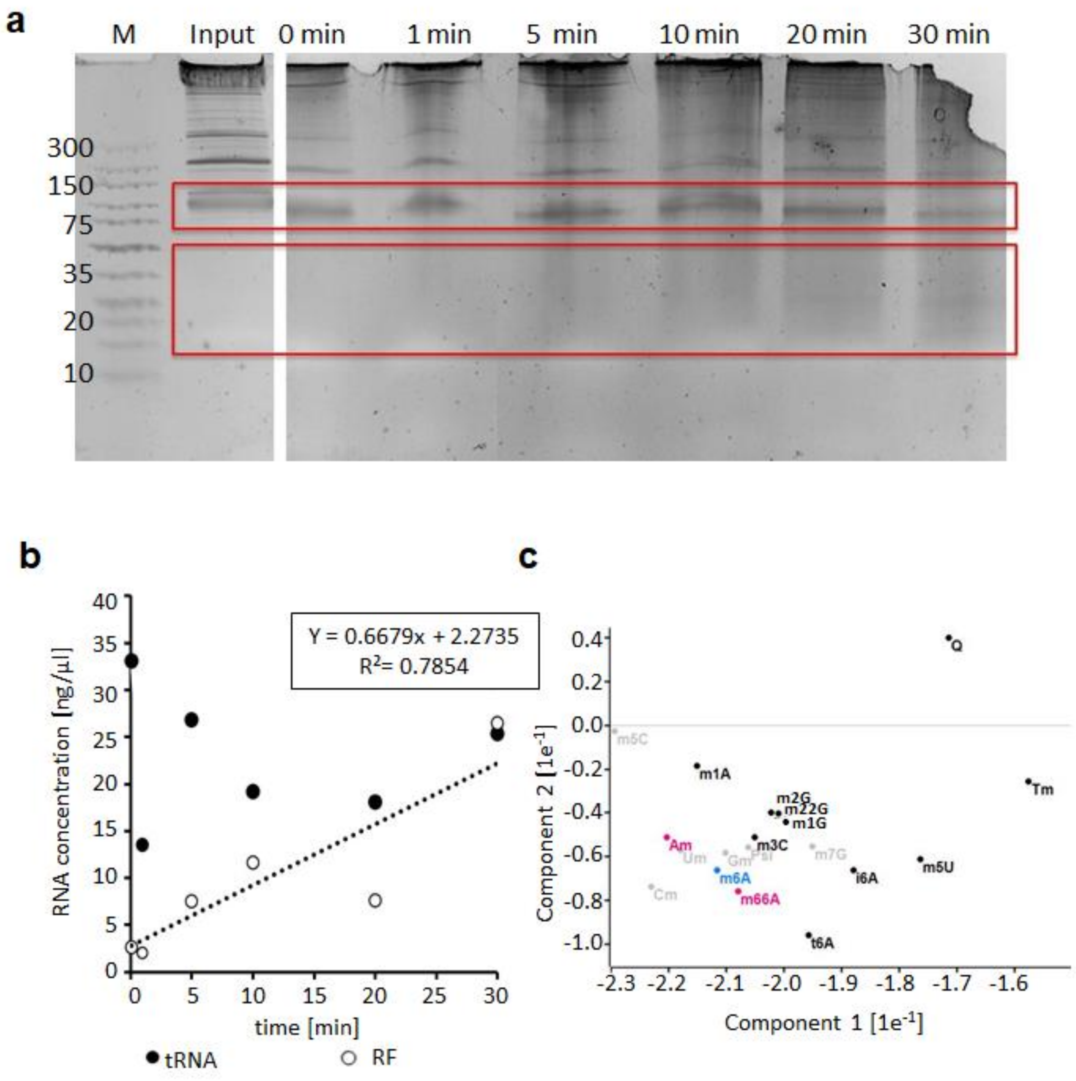

Supplementary Figure S2. Preparative isolation of *in vitro* degraded RNA. Aliquots of the degradation mix were quenched as described in the methods section, and the precipiatated RNA was submitted to a 10% PAGE, from which tRNA and RF fractions were excised (red boxes in panel a) and eluted. The yields indicated in (b) relate to a total volume of 100 µL. Panel (c) shows a zoom into the PCA of RF modifications from figure 2F.

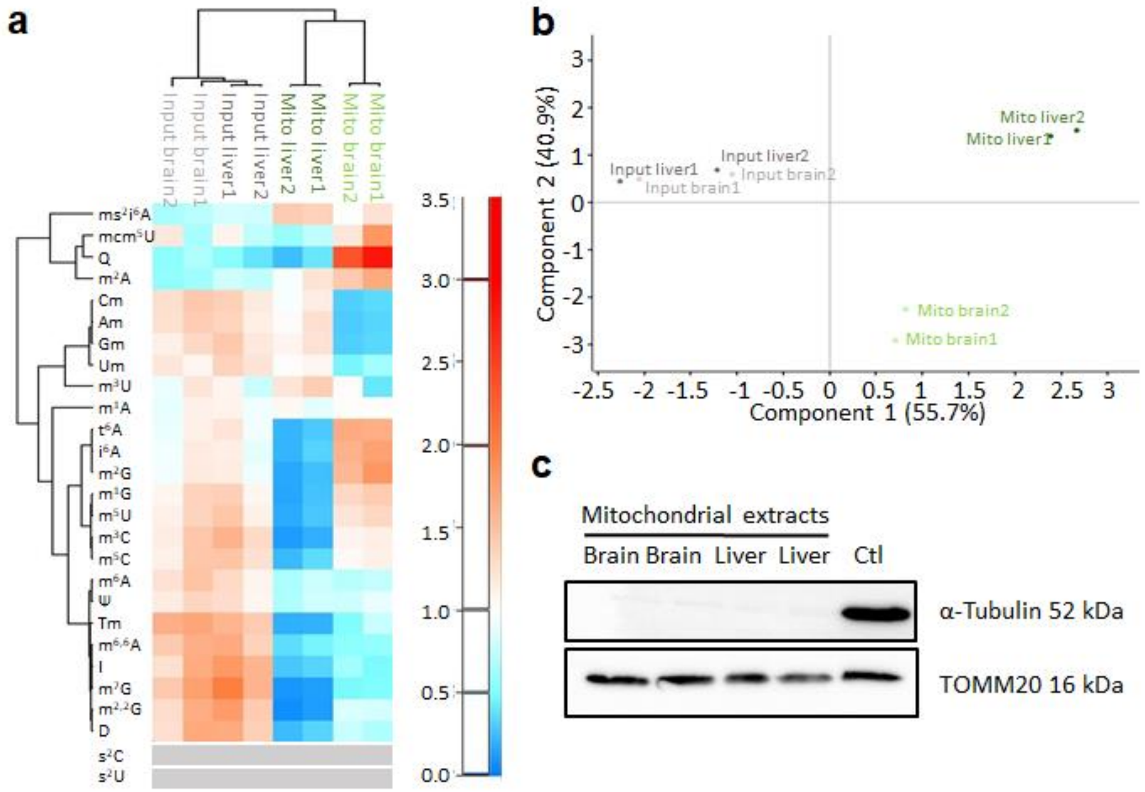

Supplementary Figure S3. Enrichment of mitochondria tracked by modification profiles. (a) Heatmap of modification content of input (total RNA from liver and brain) *versus* RNA of isolated mitochondria from liver and brain tissue. Input brain: light grey, input liver: dark grey, mitochondrial extracts from brain tissue: light green, mitochondrial extracts from liver: dark green. n=2 biologial replicates, n=2 technical replicates. (b) Principle component analysis of the samples shown in (a) with identical color code. (c) Western Blot analysis of mitochondrial extracts versus whole mouse brain protein extracts (Ctl). α-Tubulin is not present in the mitochondrial extracts as it is a cytosolic protein and thereby confirms the purity the mitochondrial preparation. The mitochondrial marker TOMM20 is visible in the mitochondrial extracts as it is found in the mitochondrial outer membrane.

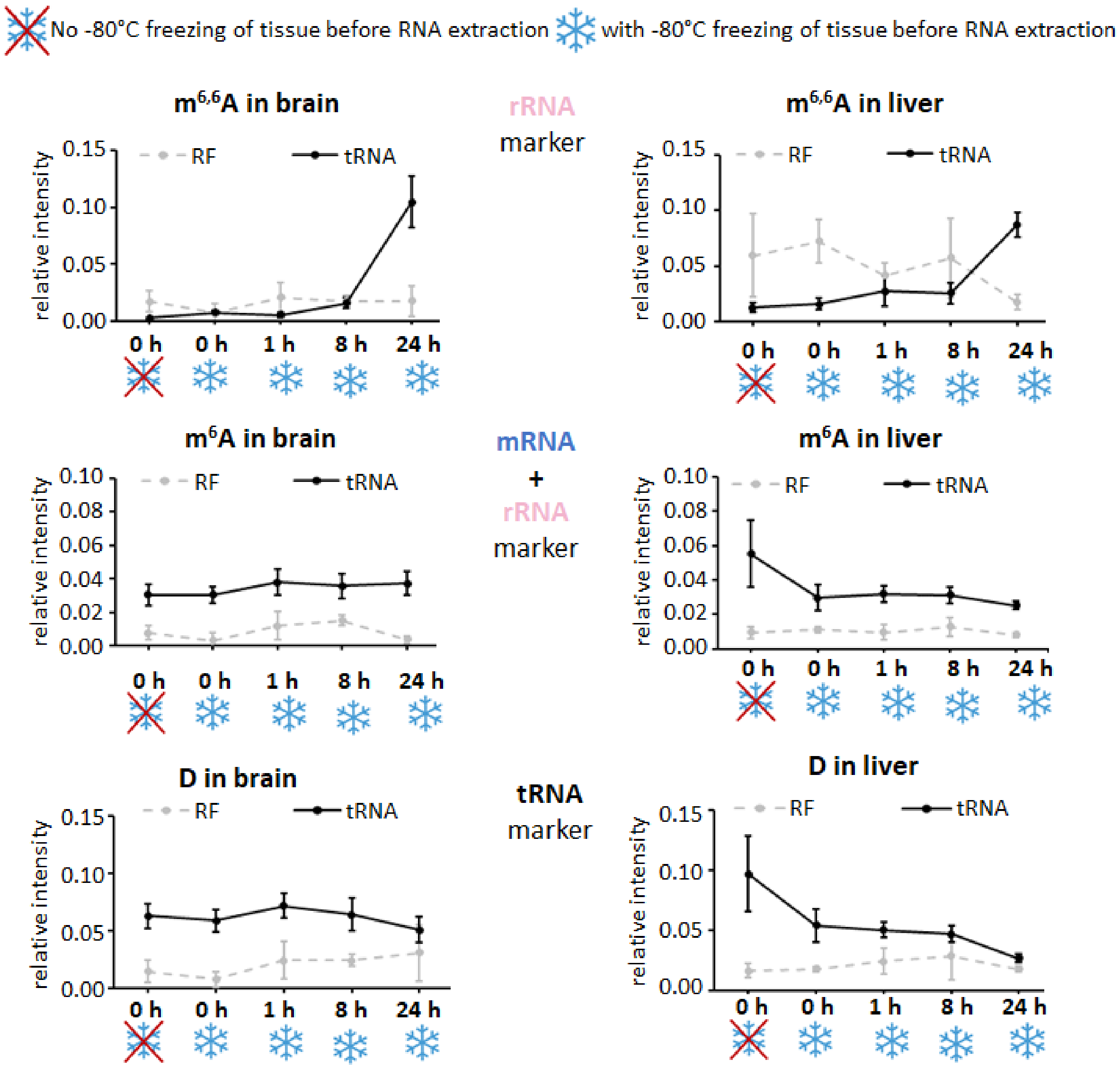

Supplementary Figure S4. Relative abundance of selected RNA modifications over time in tRNA & RF fractions during morgue experiment.

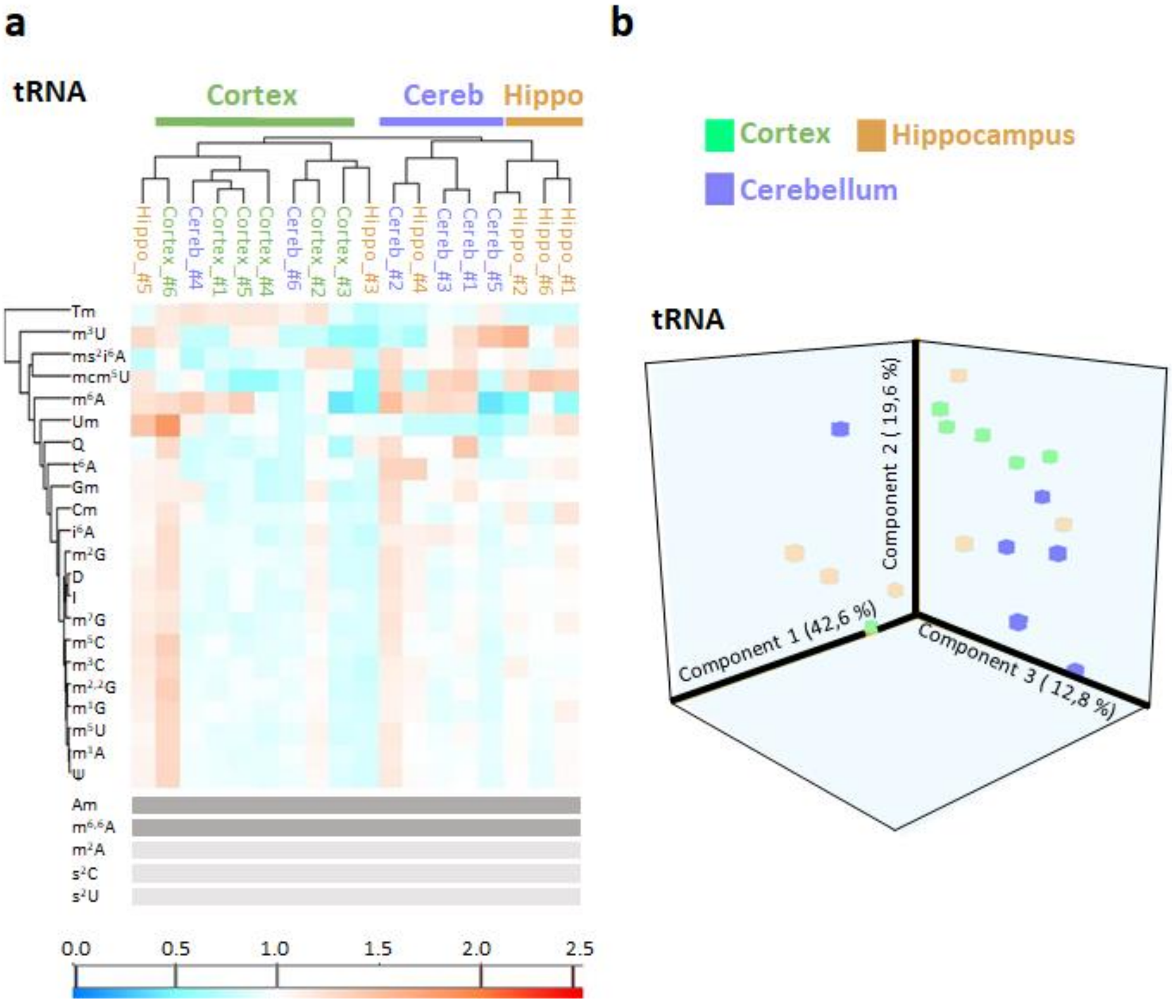

Supplementary Figure S5. Comparison of different mouse brain tissues (cohort 2). (a) Heatmap displaying tRNA and RF modification content of different brain areas from mice. n=4 biological replicates. (b) Principal component analysis of brain area samples from (a). rRNA modifications in dark grey were omitted from the analysis. Modifications with signals below 5x S/N across all samples are represented by light grey bars.
